# Understanding the role of cessation fatigue in smoking relapse: Findings from the International Tobacco Control Four Country Smoking and Vaping Survey

**DOI:** 10.1111/add.70196

**Published:** 2025-10-15

**Authors:** Hua‐Hie Yong, Ron Borland, Michael Le Grande, Claire Chia‐Yu Hu, Coral Gartner, Andrew Hyland, Kenneth Michael Cummings

**Affiliations:** ^1^ School of Psychology Deakin University Burwood Australia; ^2^ School of Public Health The University of Queensland Brisbane Australia; ^3^ Department of Health Behavior Roswell Park Comprehensive Cancer Center Buffalo NY USA; ^4^ Department of Psychiatry and Behavioral Sciences Medical University of South Carolina Charleston SC USA

**Keywords:** abstinence self‐efficacy, cessation fatigue, former smokers, relapse risk, urges to smoke, vaping

## Abstract

**Background and Aim:**

Relapse risk among people who formerly smoke is influenced by task difficulty. Cessation fatigue (CF) may be a better predictor than measures such as reported strength of urges to smoke (SUTS) and abstinence self‐efficacy (ASE). It may also be affected by quit length and use of other nicotine products. The current study investigated whether post‐quitting CF predicts higher relapse risk, its predictive utility relative to ASE and SUTS and whether the CF‐relapse prediction was moderated by time since quitting.

**Design:**

Data drawn from longitudinal cohort surveys conducted between 2016 and 2022 of the International Tobacco Control Four Country Smoking and Vaping Survey.

**Setting:**

Canada, the United States, England and Australia.

**Participants:**

People aged 18 + years who formerly smoked (*n* = 1914).

**Measurements:**

Generalised estimating equations logistic regression models were used to test for associations and moderation.

**Findings:**

In separate individual analyses, CF, ASE and SUTS were statistically significant independent relapse predictors; however, when analysed together, CF was the only statistically significant relapse predictor [moderate CF: odds ratio (OR) = 1.64, 95% confidence interval (CI) = 1.21–2.23, *P* = 0.002; high CF: OR = 1.81, 95% CI = 1.07–3.07, *P* = 0.027) on top of continuing main effects of vaping and time since quitting, but time since quitting was not a moderator.

**Conclusions:**

Cessation fatigue appears to predict smoking relapse risk better than other measures related to task difficulty and does so independently of vaping and time since quitting, which are both protective.

## INTRODUCTION

Tobacco smoking is a leading cause of preventable death and disease [1]. Most people who smoke intend to quit and approximately 40% make a quit attempt in a given year [2], but 95% of unassisted attempts ultimately end in relapse [3]. Even when evidence‐based treatments are used, relapse rates remain high [4]. To inform the development of relapse prevention interventions, a better understanding of factors that influence relapse is needed.

Relapse is generally defined as the return to smoking after a period of abstinence [5]. Relapse is a dynamic process of which many factors can influence [6]. Relapse is theorised to arise from failure of self‐control in the face of withdrawal symptoms, temptations and stressors [7]. While exercising self‐control can exhaust self‐management functions, this cannot explain relapse beyond the early days of behaviour change when self‐control is most needed and, therefore, the potential for exhaustion is real [7, 8]. Self‐control, like most self‐regulatory activity, is a limited resource and extensive periods of activity are experienced as effortful [8, 9]. Piasecki *et al*. [6] proposed a factor capturing diminished motivation to maintain self‐regulation because of the cumulative toll of cessation efforts, which they termed cessation fatigue (CF). As they theorize it, CF leads to decreases in self‐efficacy and motivation. CF is postulated to influence relapse even after withdrawal symptoms and conditioned responses fade based on the theory that it is generated, at least in part, by the continuing need to divert efforts to maintain goal pursuit (staying quit) [8].

Using an experimental design, Heckman *et al*. [7] found self‐control depletion, a key indicator of CF, decreased latency to smoke. Heckman *et al*. [10, 11] also explored possible aspects of CF using a scale with three subscales (emotional exhaustion, pessimism and devaluation) in people who recently quit smoking. Of the three subscales, they found emotional exhaustion had the clearest relationships with higher withdrawal symptoms and life stress and also showed the strongest predictive validity.

Heckman *et al*. [12] subsequently conducted a cross‐sectional study to explore the construct validity of a single‐item measure of CF theorised to tap into emotional exhaustion because of quitting smoking. They found higher post‐quitting CF was associated with shorter duration of smoking abstinence, which could be because CF causes relapse, but the study did not directly test this. Nevertheless, the finding is consistent with past research showing that CF is not stable over the course of quitting. Studies in the early days of a quit attempt suggest fatigue grows up to approximately 6 weeks into a quit attempt (among those remaining abstinent), then plateaus [11, 13, 14] or declines [12] and is mitigated by use of nicotine replacement therapy [15].

### CF as a predictor of relapse

Several studies have shown that CF predicts relapse. Liu *et al*. [14] studied among adults who currently smoked and who were motivated to quit and found higher CF predicted lower abstinence (i.e. higher relapse risk) at 6‐month follow‐up. Heckman *et al*. [11] examined the predictive utility of CF scale among people who currently smoked and those who had recently quit. They found emotional exhaustion predicted longer delay to achieving abstinence and higher relapse risk over a 12‐week study period [11]. Heckman *et al*. [12] subsequently used a single item measure of CF focussed on emotional exhaustion among people who recently quit smoking surveyed as part of the International Tobacco Control (ITC) study and found higher post‐quitting CF as measured by emotional exhaustion was prospectively associated with shorter duration of smoking abstinence at the next survey wave.

Using more recent ITC data, Cea *et al*. [16] found higher CF predicted a lower rate of sustained abstinence at the 2‐year follow‐up among those who made a quit attempt, but this effect was not independent of other quitting difficulty measures, such as urges to smoke and quit self‐efficacy, at least when assessed pre‐quitting [16]. These findings suggest that the emotional exhaustion component of CF is a useful predictor of both cessation and relapse.

To date, no study has attempted to explore whether CF adds predictive power to relapse likelihood when measured post‐quitting. It is plausibly related to other measures related to dependence or task difficulty such as strength of urges to smoke (SUTS) and abstinence self‐efficacy (ASE). SUTS is a theorized determinant of CF regardless of the model of fatigue adopted [6], and therefore, we might expect CF to be a stronger predictor of relapse than SUTS. CF itself is theorized to negatively affect ASE, but it is less certain that ASE would be a better predictor, even though conceptually it includes an assessment of commitment to persist. One characteristic of ASE is that typically it has a positive bias (‘I keep telling myself that I can, right to the point where I give up’), which might make it less predictive.

If CF is genuinely self‐control depletion, it should dissipate as SUTS become less frequent over the course of quitting (i.e. the demand reduces), and therefore, be most predictive in the early months of a quit attempt. Alternatively, if CF measures psychological exhaustion, then it could build up over time until ‘not‐smoking’ becomes fully habitual, so it might remain predictive over extended periods [8].

As nicotine vaping can help reduce nicotine withdrawal symptoms including craving [17], vaping may decrease self‐control depletion, resulting in lower CF, and therefore, may reduce relapse risk. Given a systematic review found mixed evidence for vaping status affecting relapse risk [18], and two studies yielded inconsistent findings on vaping in association with CF [11, 12], future research is needed to understand the influence on relapse of any ongoing use of nicotine vaping products post‐quitting smoking, which is expected to make the task of abstaining easier. Therefore, there is a need to clarify the extent to which CF's effect on relapse might be confounded by nicotine vaping product use.

### Study aims and hypotheses

The aims are: (a) to confirm that higher CF predicts relapse; (b) to explore whether CF is a better or an additional predictor to other measures of task difficulty (SUTS and ASE); (c) to explore whether CF's predictive effect is independent of time‐since‐quitting and any ongoing use of nicotine vaping products; and (d) to examine whether time‐since‐quitting moderates the predictive effect of CF. We hypothesised that CF would predict relapse and that it would be a stronger predictor of relapse than either SUTS or ASE. Further, its predictive power would be weaker for long‐term quitters.

## METHODS

### Study design

Data was sourced from the International Tobacco Control Four Country Smoking and Vaping (ITC 4CV) Survey, a cohort study of adults age 18+ years who have ever smoked and/or vaped in the past 2 years, conducted in Canada, the United States, England and Australia to explore the relationship between smoking and vaping, as well as to inform policies on tobacco and nicotine use [19]. Specifically, data collected in wave 1 (between July and November 2016), wave 2 (between February 2018 and July 2018), wave 3 (between February 2020 and June 2020) and wave 4 (between August 2022 and December 2022) were used. Participants were recruited from commercial online survey firms using a combination of probability and non‐probability sampling. Those lost to attrition were replenished at each follow‐up wave. To ensure sample representativeness, quotas were applied and sampling weights constructed based on respective country national benchmark figures for sex, age and geographical location. The survey took approximately 45 minutes to complete and participants were remunerated. The study procedures and materials, including the survey questionnaire, were approved by the research ethics committee at the University of Waterloo and approval was ratified by ethics committees at relevant institutions in each country. The full methodology of the ITC 4CV Survey has been published elsewhere [20, 21, 22].

### Sample characteristics

The current study included participants who had quit smoking before waves 1, 2 or 3 and who completed at least one of three baseline‐to‐follow‐up wave pairs across the four waves (i.e. waves 1–4). The analytic sample consisted of 1914 individuals providing 2715 observations across the three wave‐pairs in total. Those who were quit at multiple waves contributed multiple response sets (1260 participants contributed only one set, 507 two sets and 147 three sets across the four waves). Frequencies and percentages for sample characteristics are presented in Table 1. Across the three wave‐pairs, at baseline approximately 60% of the sample had been quit for a year or more and 44% were vaping.

**TABLE 1:** Sample characteristics (*n* = 2715 observations and *N* = 1914 unique participants)*.*

Variables	Observation level, *n*	%	Individual level, *N*	%
Gender				
Female	1444	46.8	878	45.9
Male	1271	53.2	1036	54.1
Age group, y				
18–24	129	4.8	108	5.6
25–39	570	21.0	429	22.4
40–54	775	28.6	544	28.4
55 and up	1241	45.6	833	43.5
Country				
Canada	834	30.7	609	31.8
United States	849	31.3	602	31.5
England	645	23.8	434	22.7
Australia	387	14.2	269	14.1
Ethnicity				
Majority	2408	88.7	1688	88.2
Minority	307	11.3	226	11.8
Education				
Low	772	30.2	550	28.7
Moderate	1153	41.1	828	43.3
High	768	28.6	524	27.4
Missing information	22	0.8	12	0.6
Income				
Low	600	22.1	438	22.9
Moderate	746	27.4	524	27.4
High	1213	44.7	844	44.1
Missing	156	5.8	108	5.6
Baseline time quit				
1–3 months	376	13.9	329	17.2
4–6 months	296	10.9	270	14.1
7–12 months	413	15.2	379	19.8
1–2 y	808	29.7	694	36.3
>2 y	822	30.3	242	12.6
Baseline current vaping status				
None	1522	56.1	1116	58.3
Non‐daily	310	11.4	235	12.3
Daily	883	32.5	563	29.4

*Note*: Percentages are based on unweighted data.

Abbreviations: *n*, number of observations; *N*, number of unique individuals.

### Measures

#### Predictor variables

Baseline CF was assessed by the question: ‘To what extent are you tired of trying to stay quit?’ [14]. Responses ranged from 1 (not at all tired) to 5 (extremely tired), but were positively skewed, hence, were recoded into three categories (1, not tired; 2, somewhat tired; and 3, very tired) for analysis. Do not know responses were coded as missing (*n* = 72; 2.7%).

Baseline SUTS was assessed by asking: ‘In general, how strong have urges to smoke been in the last 24 hours?’ (0, I have not felt the urge to smoke in the last 24 hours; 1, slight; 2, moderate; 3, strong; 4, very strong, 5, extremely strong; and do not know). For analysis, responses were recoded into: no urges, slight/moderate and strong/very/extremely strong. Do not know responses were recoded as missing (*n* = 11, 0.4%).

Baseline ASE was assessed using the question: ‘How confident are you that you will remain a non‐smoker?’ (1, not at all confident; 2, slightly; 3, moderately; 4, very confident; 5, extremely confident; and do not know). For analysis, responses were recoded into three categories (not at all, slightly/moderately and very/extremely confident). Do not know responses were recoded as missing (*n* = 26, 0.9%).

Baseline time‐since‐quitting was assessed by asking about number of days since quitting smoking and responses were recoded into categories for analysis to ensure sufficient cell sizes: <3 months, 4 to 6 months, 7 to 12 months, 1 to 2 years and >2 years.

Baseline vaping status was assessed based on responses to three questions: everyone was asked: ‘Have you ever used an e‐cigarette or vaping device?’ and if ‘yes’ to ever use, were asked: ‘How often do you currently use e‐cigarettes or vaping devices?’, and if ‘no’ to current use, were asked: ‘At the time that you were using e‐cigarettes or vaping devices most often, how often did you use them?’ For analysis, responses were recoded into: none (never/past non‐daily or daily vaping), current non‐daily vaping or current daily vaping.

#### Outcome variable

Relapse was assessed at follow‐up based on self‐reported smoking status. Relapse was identified among those quit at baseline who reported they were smoking at follow‐up.

### Control variables

These included baseline socio‐demographic variables, such as gender (male or female with those who indicated other coded based on sex at birth), age group (18–24, 25–39, 40–54 and 55+), country (Canada, United States, England and Australia), ethnicity (identified majority vs. minority group), education and income levels. To facilitate cross‐country comparison, education was categorised using a tertile split within each country into low, moderate or high to equate them across countries [23]. Income was similarly categorised within each country as low, moderate, or high, with refused or do not know responses combined into a fourth category (no answer). The current study additionally controlled for vaping status and time since quitting described above, with the latter also tested as a moderator variable.

### Analyses

All analyses were conducted using Stata (Version 18), with statistical significance set at *P* < 0.05. As an initial exploration of key variables, frequencies and percentages were calculated and a contingency table was constructed summarising relationships of the three measures of task difficulty (CF, SUTS and ASE) with relapse, overall and by vaping status and time since quitting. To account for the correlated nature of the data across waves and to maximise the statistical power, generalised estimating equations (GEE) logistic regression models were used to examine the predictive association. As relapse is coded as a binary outcome variable, parameter estimates were obtained using the logit link function with robust variance and an unstructured correlation structure for the working correlation. GEE modelling was conducted in stages. First, preliminary correlational analysis indicated that the three measures of task difficulty were moderately correlated with each other (see Table S1) and to avoid multicollinearity issue, the GEE modelling was conducted separately in turn for CF, SUTS and ASE, along with time since quitting and vaping status, to determine their individual association with relapse while controlling for socio‐demographics. Second, modelling was repeated for each of the three task difficulty measures while also controlling for time since quitting and vaping status. Third, modelling was then conducted jointly between CF and either SUTS or ASE, while controlling for socio‐demographics, time since quitting and vaping status, to determine their relative importance as a relapse predictor. To test whether baseline time since quitting moderated the relationship between CF and relapse, a ‘CF × time since quitting’ interaction term was added into the model for this purpose. OR and 95% CI were reported. The analysis was not pre‐registered and the results should be considered exploratory.

## RESULTS

### Descriptive statistics

At baseline across all wave pairs, 28.4% of the study sample reported experiencing CF, 94.3% were at least moderately confident of being able to stay quit and 28.6% reported experiencing urges to smoke at least slightly in the last 24 hours. By follow‐up, 9.2% had relapsed. As expected (see Tables S1 and S2), CF and SUTS were positively correlated with each other (r = 0.42, *P* < 0.001) and both negatively correlated with ASE (r = −0.42 and −0.38, respectively, both *P* < 0.001). Both CF and SUTS were lower while ASE was higher with time since quitting and similar pattern of associations were found with vaping use for these task difficulty measures (see Table S2). Table 2 shows the percentage relapsing between surveys for the three task difficulty measures, overall and separately by vaping status and time since quitting at baseline.

**TABLE 2:** Percentages relapsed by difficulty measures, overall and by vaping status and time since quitting at baseline.

		Column % total	Percentages relapsed at follow‐up				
			Overall *n* = 2689	Baseline vaping status		Baseline time since quitting	
				Not vape *n* = 1503	Vape *n* = 1186	Quit <1 y *n* = 1069	Quit 1+ y *n* = 1620
Difficulty measures	Overall (row %)		9.2	11.9	5.8	16.6	4.4
Cessation fatigue	Not tired	71.4	6.8	8.8	4.5	13.6	3.6
	Moderate	23.6	14.5	17.7	9.4	20.2	7.2
	Very tired	4.8	18.8	24.1	10.2	23.0	9.8
Urges to smoke	No urges	71.4	6.9	9.2	4.3	13.1	4.0
	Slight/moderate	26.0	14.8	17.6	10.0	21.1	5.9
	Strong urges	2.6	18.3	23.4	8.3	22.6	5.6
Abstinence self‐efficacy	Not/slightly confident	5.7	17.7	24.1	9.1	19.8	11.9
	Moderate/very	44.4	12.1	14.2	8.8	18.1	6.1
	Extremely confident	49.9	5.6	7.9	3.3	12.9	2.9

*Note*: *n* = total denominator (number of observations) may not add up to 100% because of missing data on variables.

### Inferential statistics

Each of the three measures of quitting task difficulty was strongly associated with relapse when controlling for demographics (see Base model in Table 3) with SUTS and CF positively and ASE negatively associated with relapse risk as expected. In addition, relapse rates declined with time since quitting at baseline and were lower in those who were vaping at baseline.

**TABLE 3:** Generalized estimating equation analysis of baseline cessation fatigue predicting smoking relapse at follow‐up (*n* = 2643; *N* = 1881).

	Base model			Final model		
	Individual predictor + demog			CF + demog + TQ + VS		
	OR	95% CI	*P*	OR	95% CI	*P*
Focal predictors						
CF						
Not tired	ref			Ref		
Moderate tired	**2.33**	1.74–3.11	<0.001	**1.64**	1.21–2.23	0.002
Very tired	**3.15**	1.93–5.12	<0.001	**1.81**	1.07–3.07	0.027
SUTS						
No urges	ref			NA		
Slight/moderate	**2.29**	1.74–3.03	<0.001	NA		
Strong	**2.92**	1.53–5.56	0.001	NA		
ASE						
Not/slightly confident	ref			NA		
Moderate/very confident	0.64	0.40–1.04	0.070	NA		
Extremely confident	**0.27**	0.16–0.45	<0.001	NA		
Other predictors						
TQ						
<3 months	ref			ref		
4–6 months	**0.62**	0.42–0.92	0.018	**0.62**	0.41–0.94	0.026
7–12 months	**0.33**	0.22–0.50	<0.001	**0.36**	0.23–0.55	<0.001
1–2 y	**0.19**	0.13–0.28	<0.001	**0.22**	0.14–0.33	<0.001
2+ y	**0.11**	0.07–0.17	<0.001	**0.14**	0.09–0.24	<0.001
VS						
None	ref			ref		
Non‐daily	**0.57**	0.36–0.91	0.018	**0.55**	0.33–0.92	0.022
Daily	**0.40**	0.29–0.57	<0.001	**0.60**	0.42–0.87	0.007

*Note*: OR adjusted for age, gender, ethnicity, income, education, country, survey wave; n/N are less than the full sample total because of missing data.

Abbreviations: ASE, abstinence self‐efficacy; CF, cessation fatigue *n*, number of observations; *N*, number of unique individuals; SUTS, urges to smoke; TQ, time since quitting; VS, vaping status.

While controlling for both time since quitting and vaping status, we found all three task difficulty measures predictive when tested separately (see Table S3). To test if SUTS was a determinant of CF, we repeated this analysis with both CF and SUTS and found that CF was the only significant predictor of the two. We then tested CF with ASE and again found CF the only significant predictor in this pair. Therefore, we consider only including CF as the best model (see Table 3 Final model). In this model, both time since quitting and vaping status retained their effect directions, with effect sizes essentially unchanged except for daily vaping, where the effect size, while still statistically significant (OR = 0.60, 95% CI = 0.42 to 0.87), was weaker than that in the base model (OR = 0.40, 95% CI = 0.29 to 0.57).

### Interactive effects

We tested for moderation of the CF‐relapse relationship by time since quitting and found this interaction was not significant (*P* = 0.436).

### Sensitivity analysis

In addition, we conducted a sensitivity analysis to confirm whether the vaping effect was more clearly because of vaping (or may have been contributed to by use of other nicotine products), we excluded the 232 cases who were using another non‐smoked nicotine product at baseline (120 who were in the never/past vaper group and 112 who were in the current daily/non‐daily vaping group) and reran the analyses. We found similar results as before with the exception that the association of CF with relapse was no longer linear; the OR of the moderately fatigued subgroup was now slightly higher than that of the highly fatigued subgroup (OR = 1.62, 95% CI = 1.15 to 2.29, *P* = 0.006 and OR = 1.38, 95% CI = 0.73 to 2.62, *P* = 0.328, respectively), but the difference was not significant (*P* = 0.522).

## DISCUSSION

This study confirmed that CF is a predictor of smoking relapse, as are SUTS and ASE, but CF was the best single predictor in the multivariable analyses. Moreover, the predictive effect of CF was largely independent of ongoing use of nicotine vaping products and independent of time since quitting, which were both inversely predictive of relapse. There were no interactive effects with time since quitting.

The finding that those who experienced CF were more likely to relapse is consistent with previous research focusing on short‐term relapse risk [11, 14]. It is also concordant with past studies, which reported that CF was associated with decreased abstinence‐related motivational engagement [10] and increased lapse behaviour [7]. Importantly, our finding of the primacy of CF over SUTS and ASE as a predictor of relapse suggests that the predictive power of SUTS is mediated through CF, but that ASE's relationship with relapse is largely because of the component of it generated by CF, which is consistent with research showing that prolonged resisting of cravings during a quit attempt undermines abstinence self‐efficacy. Further, our finding of no interactive relationship of CF with time since quitting strongly suggests it is a form of psychological exhaustion, as if it were self‐control depletion, then its relationship with relapse should decline with reduced levels of urges over time, and therefore, time since quitting. However, it remains plausible that relapse may be triggered by self‐control later in a quit attempt becoming overwhelmed by an acute craving, triggered by an external event (e.g. seeing someone smoking).

Additionally of note is the finding of current vaping being associated with lower SUTS and higher ASE as well as lower CF as expected, and the confirmation that vaping can reduce cravings to improve a person's confidence and capacity to maintain abstinence, therefore, helping to facilitate successful smoking cessation [17, 18, 24]. However, it is unclear why the differential effect between daily and non‐daily vaping largely disappeared once adjusted for CF and time since quitting.

### Clinical implications

Our findings are consistent with CF being the key determinant of relapse among measures of task difficulty, and therefore, a potential target for intervention. However, experimental studies confirming that modifying CF can reduce relapse are needed before any strong causal connections can be made.

The finding also has some implications for understanding how vaping may reduce relapse. Vaping has been demonstrated to lessen the workload associated with smoking cessation [17, 25], and it was expected to lower the drain on self‐control resources, which in turn would lower CF, and therefore, reducing relapse risk. This is what we found, but the effect was only partial suggesting that vaping may also aid relapse prevention in ways other than by reducing task difficulty and, therefore, lowering CF.

Having shown that CF is a risk factor for relapse, it may be possible to identify people who are particularly vulnerable, and therefore, require additional support. That a single‐item measure of CF showed predictive validity for relapse, and being able to do so over and above other known predictors, suggests its potential to be adopted as a screening tool to assess relapse risk. Assessment of CF could be incorporated in routine health checks for people who have quit smoking, particularly recently, with relapse prevention interventions provided where CF was high.

### Limitations and future research

These study findings should be interpreted with caution because of several limitations, which must be acknowledged. First, our sample had high attrition at follow‐up (i.e. 61% were lost at follow‐up), contributing to a non‐representative study sample that may affect the generalizability of our findings. For example, there were systematic differences in socio‐demographic characteristics between participants who were retained versus those who were lost to follow‐up (i.e. retained participants were older and less likely to be from England, but more likely to be from identified majority group and from low education background) (see Table S4). In addition, our sample were skewed toward people who had quit long‐term (i.e. few people who had only quit very recently), which could explain the low relapse rate in our study and also potentially making nicotine withdrawal, an important predictor of early relapse, less relevant. Future replication research is needed to determine the robustness of our findings. Second, CF was measured with a single item, which may not have adequately captured all aspects of the latent construct. Third, the long interval of 2 years between baseline and follow‐up meant that it was not possible to establish a precise sequence of events. It is not clear when relapse occurred or whether CF was present at the moment of relapse. Future research could measure key variables at more frequent time points to isolate specific effects on the temporal dynamics of relapse. Fourth, the extent to which CF might mediate the effects of SUTS and/or ASE on relapse was not explored. Future research could undertake mediation analysis to provide insights into the pathway of influence of these measures on relapse.

## CONCLUSIONS

This study confirms that CF is a useful predictor of relapse and is predictive independent of baseline time since quitting and largely independent of vaping post‐quitting. It would appear to be a better predictor than reported SUTS and ASE. This implies the potential value of assessing CF in clinical setting to identify those who may be at heightened risk and may, therefore, require personalised treatment. The discovery that time since quitting does not weaken the influence of CF on relapse highlights the need to screen for fatigue in individuals regardless of time since quitting. This study supports a psychological fatigue rather than self‐control depletion model of CF for influencing longer term relapse.

## AUTHOR CONTRIBUTIONS

**Hua‐Hie Yong** and **Ron Borland**: Conceptualization (lead); data curation (lead); formal analysis (supporting); investigation (equal); visualization (lead); writing—original draft (equal); writing—review and editing (equal). **Michael Le Grande**: Formal analysis (lead); writing—review and editing (equal). **Claire Chia‐Yu Hu**: Writing—original draft (equal); writing—review and editing (equal). **Coral Gartner**, **Andrew Hyland** and **Kenneth Michael Cummings**: investigation (supporting); writing—review and editing (equal). All authors contributed to and have approved the final manuscript.

## DECLARATION OF INTERESTS

K.M.C. has in the past and continues to serve as a paid witness in litigation filed against cigarette manufacturers. None of the other authors has any conflict of interest to declare.

## ETHICS STATEMENT

The survey protocols and all materials, including the survey questionnaires, were cleared for ethics by Research Ethics Office, King's College London, United Kingdom (IRB RESCM‐17/18‐2240); Research Ethics Board, University of Waterloo, Canada (REB20803/30570, REB20803/30878); Human Research Ethics, Cancer Council Victoria, Australia (IRB HREC 1603); Deakin University, Australia (DUHREC2018‐346); and the University of Queensland, Australia (2 016 000 330/HREC1603). Research ethics committee reviews were waived at the University of Melbourne (Melbourne, Australia) and at Medical University of South Carolina because of minimal risk.

## Supporting information


**Table S1.** Correlation matrix among task difficulty measures, time since quitting and vaping status (n = 2715).
**Table S2.** Task difficulty measures by vaping status and time since quitting at baseline.
**Table S3.** Generalized Estimating Equation Analysis Predicting Smoking Relapse at Follow‐up (n = 2643; N = 1881).
**Table S4.** Characteristics of the retained versus lost sample.

## Data Availability

In each country participating in the International Tobacco Control Policy Evaluation (ITC) Project, the data are jointly owned by the lead researcher(s) in that country and the ITC Project at the University of Waterloo. Data from the ITC Project are available to approved researchers 2 years after the date of issuance of cleaned data sets by the ITC Data Management Centre. Researchers interested in using ITC data are required to apply for approval by submitting an International Tobacco Control Data Repository (ITCDR) request application and subsequently to sign an ITCDR data usage agreement. The criteria for data usage approval and the contents of the data usage agreement are described online (http://www.itcproject.org).
