# Supplementary material for: Understanding the role of cessation fatigue in smoking relapse: Findings from the International Tobacco Control Four Country Smoking and Vaping Survey
Source: Addiction. 2025 Oct 15;121(2):340–8. doi: 10.1111/add.70196 (PMC12779587; doi:10.1111/add.70196)
Supplement: Supplementary file 1 — Table S1. Correlation matrix among task difficulty measures, time since quitting and vaping status (n = 2715). Table S2. Task difficulty measures by vaping status and time since quitting at baseline. Table S3. Generalized Estimating Equation Analysis Predicting Smoking Relapse at Follow‐up (n = 2643; N = 1881). Table S4. Characteristics of the retained versus lost sample. [file ADD-121-340-s001.docx]

**Supplementary Table S1.** *Correlation matrix among task difficulty measures, time since quitting and vaping status (n=2715)*

|  | CF | SUTS | ASE | TQ |
| --- | --- | --- | --- | --- |
| Cessation fatigue (CF)  Urges to smoke (SUTS)  Abstinence self-efficacy (ASE)  Time since quitting (TQ)  Vaping status (VS) | --  0.42  -0.43  -0.27  -0.10 | --  --  -0.37  -0.31  -0.09 | --  --  --  0.37  0.13 | --  --  --  --  0.24 |

Note: all correlations in the table are significant at p<.001; All variables based on the full expanded version with ‘don’t know’ recoded to missing; n=number of observations.

**Supplementary Table S2.** *Task difficulty measures by vaping status and time since quitting at baseline.*

| **Task difficulty measures:** | | **Baseline current vaping status (VS)**  **n=2643** | | | **Baseline time since quitting (TQ)**  **n=2643** | | | | |
| --- | --- | --- | --- | --- | --- | --- | --- | --- | --- |
|  |  | Not currently vaping  n=1487 | Vape nondaily  n=302 | Vape daily  n=854 | 1-3m  n=365 | 4-6m  n=284 | 7-12m  n=398 | 1-2 yrs  n=793 | 2+ yrs  n=803 |
| **Cessation fatigue (CF)** | Not tired | 68.9 | 56.6 | 81.0 | 48.2 | 61.6 | 64.8 | 74.5 | 85.6 |
|  | Moderate | 25.8 | 37.1 | 15.5 | 39.5 | 33.1 | 28.4 | 22.7 | 12.1 |
|  | Very tired | 5.3 | 6.3 | 3.5 | 12.3 | 5.3 | 6.8 | 2.8 | 2.4 |
| **Urges to smoke (SUTS)** | No urges | 67.7 | 61.8 | 81.1 | 41.7 | 58.8 | 69.5 | 75.6 | 86.3 |
|  | Slight/Moderate | 29.2 | 35.0 | 17.4 | 51.3 | 36.8 | 27.1 | 23.3 | 12.6 |
|  | Strong urges | 3.1 | 3.2 | 1.6 | 7.0 | 4.4 | 3.4 | 1.1 | 1.1 |
| **Abstinence Self-Efficacy (ASE)** | Not/slightly confident | 5.8 | 11.0 | 3.7 | 15.4 | 10.0 | 6.1 | 3.4 | 1.8 |
|  | Moderate/very | 49.3 | 51.1 | 33.8 | 60.8 | 57.4 | 51.5 | 44.2 | 29.2 |
|  | Extremely confident | 44.9 | 37.9 | 62.6 | 23.8 | 32.7 | 42.4 | 52.4 | 68.9 |

Note: n=number of observations, less than full sample total due to missing data

Supplementary Table S3. Generalized Estimating Equation Analysis Predicting Smoking Relapse at Follow-up (n=2643; N=1881).

|  | Base Model 1  Individual  predictor  + demog | |  | Model 2A  CF  + demog  + TQ + VS | |  | Model 2B  SUTS  + demog  + TQ + VS | |  | Model 2C  ASE  + demog  + TQ + VS | |  | Model 3  CF + SUTS  + demog  + TQ + VS | |  | Model 4  CF + ASE  + demog  + TQ + VS | |  |
| --- | --- | --- | --- | --- | --- | --- | --- | --- | --- | --- | --- | --- | --- | --- | --- | --- | --- | --- |
|  |  |  |  |  |  |  |  |  |  |  |  |  |  |  |  |  |  |  |
|  | OR | 95% CI |  | OR | 95% CI |  | OR | 95% CI |  | OR | 95% CI |  | OR | 95% CI |  | OR | 95% CI |  |
| **Focal predictors:** |  |  |  |  |  |  |  |  |  |  |  |  |  |  |  |  |  |  |
| Cessation fatigue (CF) |  |  |  |  |  |  |  |  |  |  |  |  |  |  |  |  |  |  |
| Not tired | ref |  |  | ref |  |  | NA |  |  | NA |  |  | ref |  |  | ref |  |  |
| Mod tired | 2.33*** | 1.74, 3.11 |  | 1.64** | 1.21, 2.23 |  | NA |  |  | NA |  |  | 1.50* | 1.07, 2.10 |  | 1.44* | 1.03, 2.00 |  |
| Very tired | 3.15*** | 1.93, 5.12 |  | 1.81* | 1.07, 3.07 |  | NA |  |  | NA |  |  | 1.74* | 1.00, 3.04 |  | 1.63 | 0.95, 2.80 |  |
| Urges to smoke (SUTS) |  |  |  |  |  |  |  |  |  |  |  |  |  |  |  |  |  |  |
| No urges | ref |  |  | NA |  |  | ref |  |  | NA |  |  | ref |  |  | NA |  |  |
| Slight/moderate | 2.29*** | 1.74, 3.03 |  | NA |  |  | 1.44* | 1.07, 1.94 |  | NA |  |  | 1.25 | 0.90, 1.73 |  | NA |  |  |
| Strong | 2.92** | 1.53, 5.56 |  | NA |  |  | 1.54 | 0.77, 3.10 |  | NA |  |  | 1.07 | 0.50, 2.29 |  | NA |  |  |
| Abstinence SE (ASE) | |  |  |  |  |  |  |  |  |  |  |  |  |  |  |  |  |  |
| Not/slightly confident | ref |  |  | NA |  |  | NA |  |  | ref |  |  | NA |  |  | ref |  |  |
| Mod/very confident | 0.64 | 0.40, 1.04 |  | NA |  |  | NA |  |  | 0.89 | 0.53, 1.48 |  | NA |  |  | 0.93 | 0.55, 1.57 |  |
| extremely confident | 0.27*** | 0.16, 0.45 |  | NA |  |  | NA |  |  | 0.56* | 0.32, 0.98 |  | NA |  |  | 0.64 | 0.35, 1.16 |  |
| **Other predictors:** |  |  |  |  |  |  |  |  |  |  |  |  |  |  |  |  |  |  |
| Time since quitting (TQ) |  |  |  |  |  |  |  |  |  |  |  |  |  |  |  |  |  |  |
| <3m | ref |  |  | ref |  |  | ref |  |  | ref |  |  | ref |  |  | ref |  |  |
| 4-6m | 0.62* | 0.42, 0.92 |  | 0.62* | 0.41, 0.94 |  | 0.67* | 0.45, 1.00 |  | 0.64* | 0.43, 0.96 |  | 0.64* | 0.42, 0.98 |  | 0.62* | 0.41, 0.95 |  |
| 7-12m | 0.33*** | 0.22, 0.50 |  | 0.36*** | 0.23, 0.55 |  | 0.38*** | 0.25, 0.58 |  | 0.35*** | 0.23, 0.54 |  | 0.38*** | 0.24, 0.58 |  | 0.35*** | 0.23, 0.54 |  |
| 1-2y | 0.19*** | 0.13, 0.28 |  | 0.22*** | 0.14, 0.33 |  | 0.22*** | 0.15, 0.33 |  | 0.21*** | 0.14, 0.32 |  | 0.23*** | 0.15, 0.35 |  | 0.22*** | 0.15, 0.34 |  |
| 2+y | 0.11*** | 0.07, 0.17 |  | 0.14*** | 0.09, 0.24 |  | 0.14*** | 0.09, 0.24 |  | 0.15*** | 0.09, 0.25 |  | 0.15*** | 0.09, 0.25 |  | 0.16*** | 0.10, 0.27 |  |
| Vaping status (VS) |  |  |  |  |  |  |  |  |  |  |  |  |  |  |  |  |  |  |
| Non-vaper | ref |  |  | ref |  |  | ref |  |  | ref |  |  | ref |  |  | ref |  |  |
| Non-daily vaper | 0.57* | 0.36, 0.91 |  | 0.55* | 0.33, 0.92 |  | 0.53* | 0.32, 0.87 |  | 0.56* | 0.34, 0.92 |  | 0.53* | 0.32, 0.88 |  | 0.55* | 0.33, 0.91 |  |
| Daily vaper | 0.40*** | 0.29, 0.57 |  | 0.60** | 0.42, 0.87 |  | 0.57** | 0.40, 0.82 |  | 0.59** | 0.41, 0.85 |  | 0.59** | 0.41, 0.85 |  | 0.61** | 0.42, 0.88 |  |
| Age group |  |  |  |  |  |  |  |  |  |  |  |  |  |  |  |  |  |  |
| 18-24 | NS |  |  | ref |  |  | ref |  |  | ref |  |  | ref |  |  | ref |  |  |
| 25-39 | NS |  |  | 2.22 | 0.99, 4.96 |  | 2.10 | 0.95, 4.66 |  | 2.15 | 0.97, 4.81 |  | 2.17 | 0.97, 4.87 |  | 2.26* | 1.00, 5.11 |  |
| 40-54 | NS |  |  | 1.85 | 0.84, 4.12 |  | 1.82 | 0.83, 4.00 |  | 1.90 | 0.86, 4.21 |  | 1.81 | 0.81, 4.01 |  | 1.89 | 0.84, 4.24 |  |
| 55+ | NS |  |  | 1.48 | 0.66, 3.30 |  | 1.44 | 0.65, 3.16 |  | 1.45 | 0.65, 3.23 |  | 1.43 | 0.64, 3.20 |  | 1.47 | 0.65, 3.33 |  |
| Gender |  |  |  |  |  |  |  |  |  |  |  |  |  |  |  |  |  |  |
| Male | NS |  |  | ref |  |  | ref |  |  | ref |  |  | ref |  |  | ref |  |  |
| Female | NS |  |  | 1.08 | 0.81, 1.43 |  | 1.07 | 0.81, 1.41 |  | 1.06 | 0.80, 1.41 |  | 1.08 | 0.81, 1.43 |  | 1.08 | 0.81, 1.43 |  |
| Ethnicity |  |  |  |  |  |  |  |  |  |  |  |  |  |  |  |  |  |  |
| Identified majority | NS |  |  | ref |  |  | ref |  |  | ref |  |  | ref |  |  | ref |  |  |
| Identified minority | NS |  |  | 0.98 | 0.64, 1.50 |  | 1.06 | 0.69, 1.60 |  | 1.01 | 0.66, 1.56 |  | 1.00 | 0.65, 1.54 |  | 0.99 | 0.64, 1.53 |  |
| Education |  |  |  |  |  |  |  |  |  |  |  |  |  |  |  |  |  |  |
| Low | NS |  |  | ref |  |  | ref |  |  | ref |  |  | ref |  |  | ref |  |  |
| Moderate | NS |  |  | 1.13 | 0.80, 1.59 |  | 1.14 | 0.82, 1.59 |  | 1.12 | 0.80, 1.58 |  | 1.14 | 0.81, 1.60 |  | 1.14 | 0.80, 1.61 |  |
| High | NS |  |  | 0.94 | 0.63, 1.39 |  | 0.89 | 0.61, 1.32 |  | 0.91 | 0.61, 1.35 |  | 0.91 | 0.61, 1.36 |  | 0.93 | 0.62, 1.39 |  |
| No info | NS |  |  | 0.87 | 0.10, 7.35 |  | 0.88 | 0.10, 7.45 |  | 0.96 | 0.12, 7.53 |  | 0.90 | 0.11, 7.67 |  | 0.96 | 0.12, 7.72 |  |
| Income |  |  |  |  |  |  |  |  |  |  |  |  |  |  |  |  |  |  |
| Low | NS |  |  | ref |  |  | ref |  |  | ref |  |  | ref |  |  | ref |  |  |
| Moderate | NS |  |  | 0.78 | 0.53, 1.14 |  | 0.78 | 0.54, 1.14 |  | 0.75 | 0.51, 1.09 |  | 0.78 | 0.53, 1.15 |  | 0.74 | 0.50, 1.10 |  |
| High | NS |  |  | 0.66* | 0.46, 0.94 |  | 0.69* | 0.49, 0.99 |  | 0.68* | 0.47, 0.97 |  | 0.68* | 0.48, 0.98 |  | 0.66* | 0.46, 0.95 |  |
| No info | NS |  |  | 0.63 | 0.32, 1.24 |  | 0.65 | 0.33, 1.27 |  | 0.65 | 0.33, 1.27 |  | 0.65 | 0.33, 1.28 |  | 0.64 | 0.33, 1.26 |  |
| Survey wave (year) |  |  |  |  |  |  |  |  |  |  |  |  |  |  |  |  |  |  |
| Wave 1 (2016) | NS |  |  | ref |  |  | ref |  |  | ref |  |  | ref |  |  | ref |  |  |
| Wave 2 (2018) | NS |  |  | 1.18 | 0.84, 1.65 |  | 1.23 | 0.88, 1.64 |  | 1.17 | 0.83, 1.63 |  | 1.21 | 0.86, 1.70 |  | 1.15 | 0.82, 1.62 |  |
| Wave 3 (2020) | NS |  |  | 0.98 | 0.67, 1.44 |  | 0.94 | 0.64,  1.39 |  | 0.91 | 0.62, 1.34 |  | 1.00 | 0.67, 1.47 |  | 0.95 | 0.65, 1.40 |  |
| Country |  |  |  |  |  |  |  |  |  |  |  |  |  |  |  |  |  |  |
| Canada | NS |  |  | ref |  |  | ref |  |  | ref |  |  | ref |  |  | ref |  |  |
| United States | NS |  |  | 1.14 | 0.79, 1.64 |  | 1.13 | 0.80,  1.61 |  | 1.17 | 0.82, 1.67 |  | 1.15 | 0.80, 1.66 |  | 1.15 | 0.80, 1.66 |  |
| England | NS |  |  | 1.02 | 0.68, 1.53 |  | 1.01 | 0.68, 1.50 |  | 0.98 | 0.65, 1.48 |  | 1.05 | 0.70, 1.58 |  | 1.00 | 0.66, 1.51 |  |
| Australia | NS |  |  | 1.33 | 0.88, 2.02 |  | 1.22 | 0.81, 1.85 |  | 1.32 | 0.87, 2.00 |  | 1.30 | 0.85, 1.97 |  | 1.32 | 0.86, 2.02 |  |

Note: n=number of observations; N=number of unique individuals; NS=not shown in table; NA, not applicable; **p* < .05, ***p* <.01, ****p* <.001

**Supplementary Table S4.** *Characteristics of the retained versus lost sample*

| Variables | Study sample | | Sample lost to attrition | | χ^2^ |
| --- | --- | --- | --- | --- | --- |
|  | *N* | % | *N* | % |  |
| Gender |  |  |  |  | 1.72 |
| Female | 1036 | 54.1 | 1559 | 52.2 |  |
| Male | 878 | 45.9 | 1427 | 47.8 |  |
| Age group |  |  |  |  | 207.72*** |
| 18-24 | 108 | 5.6 | 552 | 18.5 |  |
| 25-39 | 429 | 22.4 | 789 | 26.4 |  |
| 40-54 | 544 | 28.4 | 705 | 23.6 |  |
| 55+ | 833 | 43.5 | 940 | 31.5 |  |
| Country |  |  |  |  | 22.66*** |
| Australia | 609 | 31.8 | 968 | 32.4 |  |
| Canada | 602 | 31.5 | 820 | 27.5 |  |
| England | 434 | 22.7 | 836 | 28.0 |  |
| United States | 269 | 14.1 | 362 | 12.1 |  |
| Ethnicity |  |  |  |  | 23.94*** |
| Majority | 1688 | 88.2 | 2481 | 83.1 |  |
| Minority | 226 | 11.8 | 505 | 16.9 |  |
| Education |  |  |  |  | 11.67** |
| Low | 550 | 28.7 | 748 | 25.1 |  |
| Moderate | 828 | 43.3 | 1287 | 43.1 |  |
| High | 524 | 27.4 | 930 | 31.2 |  |
| Missing | 12 | 0.6 | 21 | 0.7 |  |
| Income |  |  |  |  | 4.45 |
| Low | 438 | 22.9 | 639 | 21.4 |  |
| Moderate | 524 | 27.4 | 785 | 26.3 |  |
| High | 844 | 44.1 | 1361 | 45.6 |  |
| Missing | 108 | 5.6 | 201 | 6.7 |  |

*Note*. N=Number of unique individuals; **p* < .05, ***p* <.01, ****p* <.001
